# Feasibility of Cognitive-Motor Exergames in Geriatric Inpatient Rehabilitation: A Pilot Randomized Controlled Study

**DOI:** 10.3389/fnagi.2021.739948

**Published:** 2021-11-29

**Authors:** Patrizia Altorfer, Manuela Adcock, Eling D. de Bruin, Florian Graf, Eleftheria Giannouli

**Affiliations:** ^1^Department of Health Sciences and Technology, Institute of Human Movement Sciences and Sport, ETH Zürich, Zurich, Switzerland; ^2^Division of Physiotherapy, Department of Neurobiology, Care Sciences and Society, Karolinska Institute, Stockholm, Sweden; ^3^Department of Health, OST – Eastern Swiss University of Applied Sciences, St. Gallen, Switzerland; ^4^VAMED Rehaklinik Dussnang, Clinic for Geriatric and Orthopedic Rehabilitation, Dussnang, Switzerland; ^5^Department of Sport, Exercise and Health, Division of Sports and Exercise Medicine, University of Basel, Basel, Switzerland

**Keywords:** exergaming, balance training, cognitive training, exercise, step training, older adults, fall prevention

## Abstract

**Objective:** The aim of this pilot randomized clinical trial was to test the feasibility and efficacy of an exergame-based cognitive-motor training program in geriatric inpatients.

**Methods:** The study participants were randomly allocated to either the exergame intervention group or the control group. The control group received the standard rehabilitation treatment offered in the clinic. In addition to the standard rehabilitation program, the intervention group conducted supervised exergame training on 5 days per week using the Dividat Senso, an exergame system specifically designed for older adults. The primary outcome was feasibility, as measured by e.g., adherence rate, attrition rate, occurrence of adverse events, System Usability Scale (SUS) and NASA-TLX score. Secondary outcomes included measures of physical and cognitive functioning such as comfortable walking speed, maximal walking speed, dual task walking speed, Short Physical Performance Battery (SPPB), Timed Up and Go test (TUG), Color-Word Interference test (D-KEFS), Trail Making test A and B (TMT), Go/No-Go test and Step Reaction Time test (SRTT). All secondary outcome measures were assessed pre- and post-intervention.

**Results:** Thirty-nine persons were included in the study. Average adherence rate was 99%, there were no intervention-related dropouts and no adverse events. The mean System Usability Scale (SUS) score was 83.6 and the mean NASA-TLX score 45.5. Significant time-group interaction effects were found for the dual task walking speed, the Go/No-Go test and Step Reaction Time test (SRTT).

**Conclusion:** Exergaming is a feasible, safe and effective cognitive-motor training approach in inpatient rehabilitation of geriatric patients. Incorporating exergaming in the rehabilitation program of geriatric patients offers potential to reduce fall risk factors and to increase patients’ exercise motivation and rehabilitation success.

## Introduction

The aging process is accompanied by a decline in physical and cognitive functions such as balance, gait, executive functions and psychomotor speed (Kramer et al., 1999; Park, 2000; Seidler et al., 2010; Reuter-Lorenz et al., 2015; Valenzuela et al., 2018; Cogliati et al., 2019). These declines often lead to loss of independence in daily life, restricted social participation and are major risk factors for accidental falls (Rowe and Kahn, 1997; Kramer et al., 1999; Herman et al., 2010; Khow and Visvanathan, 2017). In Switzerland, approximately 25% of people aged 65 years and older fall at least once a year (Stürze im Laufe eines Jahre, 2017). Falls in older adults often result in injuries, reduced quality of life and increased healthcare costs (Stevens et al., 2006; Hartholt et al., 2011). Frail older adults, such as people undergoing rehabilitation after a surgery or a fall, are exposed to an even higher fall risk (Kojima et al., 2015). Therefore, it is highly important to improve physical and cognitive functioning, which will in turn reduce fall risk in older adults in general but even more so in fall-prone individuals such as rehabilitation patients. A highly important physical function that is reduced with age is balance control. The balance control system consists of several components such as sensory information acquisition, information processing as well as production of an adequate motor response (Van Dieën and Pijnappels, 2017), all of which show age-related impairments. As a result, older adults frequently face difficulties in controlling their balance (Sturnieks et al., 2008), e.g., executing adequate stepping responses. Stepping responses can be divided into volitional stepping responses e.g., in order to proactively avoid an obstacle and reactive stepping responses e.g., in order to react to an external perturbation to avoid falling (Okubo et al., 2021). In older adults, this stepping capacity is frequently reduced: their reactive steps are shorter (Luchies et al., 1994) and they tend to collide the swing foot with the stance leg (Maki et al., 2000). Moreover, in fallers, the maximum step length (Cho et al., 2004; Schulz et al., 2013) and volitional stepping speed (Melzer et al., 2007) is reduced compared to non-fallers. As a result, if stepping capacity is decreased, the risk for falls increases (Okubo et al., 2017, 2021).

Training interventions that aim to improve stepping capacity were shown to reduce fall incidence (Okubo et al., 2017) and are recommended to be incorporated into fall prevention training programs (Cadore et al., 2013; Giannouli et al., 2020; Sibley et al., 2021). Step training using exergames has become an important instrument in fall prevention in the aging population (Donath et al., 2016; Choi et al., 2017). Exergames successfully combine motor and cognitive training by providing cognitively demanding games which are played by executing body movements. Increasing evidence suggests that the combination of motor and cognitive training leads to a superior effect on cognitive and dual task performance compared to motor or cognitive training alone (Levin et al., 2017; Tait et al., 2017; Raichlen et al., 2020). Simultaneous motor and cognitive training stimulates similar neurobiological processes which result in a synergistic response with higher effects on cognitive improvements (Tait et al., 2017). Therefore, such training can also be more effective in reversing the neurodegenerative consequences of aging than motor or cognitive training alone and, therefore, also decrease fall risk to a higher extent (Segev-Jacubovski et al., 2011; Schoene et al., 2014; Raichlen and Alexander, 2017). In geriatric and/or orthopedic rehabilitation however, conventional therapies merely focus on physical functions. To that end, a cognitive-motor training delivered in form of exergames could increase the efficacy of the rehabilitation by addressing also cognitive control and dual tasking. Furthermore, gamification of exercise was shown to have positive effects on training motivation (Proffitt et al., 2015) and self-efficacy (Su and Cheng, 2016) which are factors that can increase rehabilitation success (Bonnechère et al., 2016). Traditional rehabilitation strategies can be monotonous (Kamkarhaghighi et al., 2017) and exergaming offers a suitable supplement to make a rehabilitation program more entertaining and, thereby, increase the patients’ motivation to participate and adhere to their exercise routines (Kappen et al., 2019).

Exergaming interventions were shown to be a feasible and effective training approach in healthy older people; with high adherence to exergame interventions (>90%) and only rare and minor adverse events being reported (Valenzuela et al., 2018). In addition to this, exergaming interventions have positive effects on physical and cognitive functioning such as balance, functional mobility, gait and executive functions in healthy older adults (Stanmore et al., 2017; Corregidor-Sánchez et al., 2020a,b; Fang et al., 2020; Pacheco et al., 2020; Wollesen et al., 2020), as well as people suffering from chronic diseases (Bonnechère et al., 2016; Stanmore et al., 2017; Zeng et al., 2017). However, some current gaps in knowledge need to be addressed. In most of these previous studies, commercial exergame systems were used which were mostly designed for young people and recreational purposes. It can be hypothesized that an exergame system specifically designed for clinical purposes may have better effects. Furthermore, evidence regarding the feasibility of exergame training in inpatient rehabilitation settings is scarce (Knols et al., 2016; Cuevas-Lara et al., 2021).

To the best of our knowledge, this is the first study to examine the feasibility and effects of a motor-cognitive training in form of purpose-developed exergames in a geriatric inpatient rehabilitation setting. Aim of this study was to assess the feasibility and effects of such training in a geriatric inpatient rehabilitation clinic. We first hypothesized that the exergame intervention integrated in the inpatient rehabilitation program routines is feasible and safe. Secondly, we hypothesized that the effects on cognitive and physical functions will be more meaningful in the group receiving the exergame intervention compared to the group receiving the conventional rehabilitation therapy only.

## Materials and Methods

This paper presents the results of inpatient exergame rehabilitation integrated in the program from the orthopedic and geriatric rehabilitation clinic Dussnang. It is the first study within a series of studies examining feasibility of exergame training in different rehabilitation clinics and various inpatient groups. The study was approved by the cantonal ethics committee of Zurich, Switzerland (Reg.-No.: 2020-02388), and was conducted according to Good Clinical Practice (GCP) guidelines and the Declaration of Helsinki. All participants were required to give written informed consent. The study has been registered at ClinicalTrials.gov (ID: NCT04872153).

### Study Design

This is a pilot feasibility randomized clinical trial (RCT) with two arms (one intervention and one control group) adhering to the CONSORT extension for pilot and feasibility trials (Eldridge et al., 2016). The study was conducted at the geriatric and orthopedic rehabilitation clinic Dussnang during a period of 3 months (January to March 2021). Participants were randomly allocated to one of the two groups using a permuted block randomization approach. The intervention group conducted exergame-training using the Dividat Senso in addition to the conventional rehabilitation treatment while the control group received the conventional treatment only. The duration of the intervention period was adjusted to the duration of each participant’s stay at the rehabilitation clinic lasting between 2 and 3 weeks. Before beginning and after finishing the intervention or control period, a baseline (T1-meaurement) and a post measurement (T2-measurement) was conducted with each participant of both groups.

Feasibility in this study was adopted as an umbrella term encompassing adherence, attrition, patient acceptability and safety of the intervention. Adherence considered the frequency of participant attendance at the intervention sessions and the attrition considered the proportion of dropouts. Patient acceptability was assessed by enjoyment level during the intervention and two questionnaires at the end of the intervention. Safety was assessed by recording of adverse events and falls that occurred during the intervention and by two questionnaires at the end of the intervention. We a-priori adopted 15% or less attrition and 80% or more adherence as acceptable for inpatient orthopedic exergame rehabilitation (Nyman and Victor, 2011). Furthermore, patients were expected to score 70 or more points on the System Usability Scale (SUS) (Bangor et al., 2009) and 55/100 for the NASA-TLX score (Grier, 2015).

To provide an estimation of the effectiveness of the intervention for future RCTs, secondary outcomes were used to test the hypothesis of effectiveness. To ensure sufficient power, a sample size calculation was performed. Sample size calculation suggested that a total sample size of 16 participants would offer a power of 91% to correctly reject the null hypothesis. The calculation was based on the effect size (*F* = 0.4) of the interaction effect of the outcome measure Timed Up and Go (TUG) as assessed in the study by Morat et al. (2019). The exergame intervention was almost identical to the present study, however, the intervention period was more than twice as long. Because of the much shorter intervention period in this study, the sample size was aimed to be 40 allowing the detection of smaller effect sizes with sufficient power while also allowing some dropouts. Thus, a small effect size (*F* = 0.3) can be detected with a 91% chance of correctly rejecting the null hypothesis.

### Participants

At clinic entry, patients were informed in oral form about the study. Interested persons were fully informed with a detailed information sheet and gave written informed consent prior to the onset of their participation in the study. To be included in the study persons had to fulfill following criteria: (Park, 2000) in-patient stay in the orthopedic and geriatric rehabilitation clinic Dussnang, (Seidler et al., 2010) age ≥ 50 years, (Valenzuela et al., 2018) able to score ≥ 20 on the Mini Mental State Examination (MMSE), (Cogliati et al., 2019) able to provide a signed informed consent, (Reuter-Lorenz et al., 2015) physically able to stand for at least 3 min without external support (self-report). Exclusion criteria were: (Park, 2000) mobility or cognitive limitations or comorbidities which impair the ability to use the training games and overall system, (Seidler et al., 2010) conservatively treated osteoporotic fractures, (Valenzuela et al., 2018) previous or current major psychiatric illness (e.g., schizophrenia, bipolar disorder, recurrent major depression episodes), (Cogliati et al., 2019) history of drugs or alcohol abuse, (Reuter-Lorenz et al., 2015) terminal illness, (Kramer et al., 1999) severe visual (e.g., especially achromatopsia) and auditory impairments, (Khow and Visvanathan, 2017) insufficient knowledge of German to understand the instructions/games.

### Exergame Intervention

Participants allocated to the intervention group conducted a supervised motor-cognitive training by playing exergames on the Dividat Senso in addition to the standard rehabilitation treatment offered by the clinic. The Dividat Senso is a device consisting of a pressure-sensitive platform which records movement produced forces. The platform includes 20 sensors (strain gauges), five vibration motors and an LED control. It is certified as a medical device class 1 and was specifically developed for clinical use. The Dividat Senso is connected to a computer and a screen on which the stimuli appear. The Dividat exergames (Supplementary Table 1) were used which specifically target cognitive and motor functions required for activities of daily living such as executive and attentional functions and balance and coordination. The games are played by making steps in four directions (front, right, left, back) and body weight shifting. Training sessions were executed on 5 days per week and each session lasted between 10 and 15 min. During each session, the participants played between six and seven different exergames each lasting between 2 and 3 min. The participants played the same composition of games for five training sessions. After every five training sessions, new, more challenging games were introduced to the training plan. To ensure adequate training progression, personalization of the training plan was achieved on the one hand by the training software (DividatPlay), which contains an algorithm that enabled automatic, real-time adaptation of the difficulty of a training to the level of an individual participant. On the other hand, the therapist/trainer adapted the training plan (i.e., substituted single games) in case of insufficient or excessive difficulty as measured by two criteria: (Park, 2000) too low performance in a game, (Seidler et al., 2010) subjective report of the patient that the game is too difficult or too easy.

### Control Group

The patients of the control group followed the standard rehabilitation plan offered by the clinic. For each week this usually included: 3× 30 min physiotherapy, 5× 30 min group therapy (knee- / hip- or back-specific group / otago-group therapy for upper extremities), 3× 30 min walking groups (only in patients admitted for issues in the lower extremities), 3× 45 min group therapy (mindfulness therapy, medical training therapy, activating groups).

### Primary Outcomes

The primary outcome of this study was the feasibility of the Dividat Senso integrated in the rehabilitation context. For this purpose, adherence, attrition, and the number of adverse events were assessed. In addition, four questionnaires were used to assess usability and safety and filled in by the participants of the intervention group after each training (NASA-TLX, enjoyment) or at T2-measurement only (SUS, self-made questionnaire including several usability and user experience questions).

#### Adherence, Attrition and Adverse Events

Average adherence rate was calculated as the number of completed training sessions as a percentage of the maximal possible training sessions. Reasons for non-adherence were recorded in the attendance protocol. Additionally, attrition in the intervention and control group was recorded. The attrition rate was calculated as the number of participants that dropped out during the trial as a percentage of the initial sample size. Adverse events occurring during the training sessions and measurements were noted in detail by the treating therapist.

#### System Usability Scale

To assess usability of the Dividat Senso, the System Usability Scale (SUS) was used (Brooke, 1996). The SUS is often used for the evaluation of software products, websites or games/exergames. It is a validated and reliable scale and consists of ten items rated on a 5-point Likert scale ranging from 0 to 4 (Brooke, 1996; Tullis and Albert, 2008). For this study, a German translation of the SUS was used (Gao et al., 2020). Scores above 70 are regarded as “acceptable” (Bangor et al., 2009).

#### NASA Task Load Index and Enjoyment Level

The NASA Task Load Index (TLX) developed by Hart (Hart and Staveland, 1988) is a subjective assessment tool to assess workload experienced while working with a human-machine interface system. A multidimensional rating procedure is used which includes ratings on six subscales: Mental Demand, Physical Demand, Temporal Demand, Performance, Fatigue and Frustration. For each subscale, there is one question which are answered on a 20-point Likert scale ranging from “a little” until “too much.” For this study, a German translation of the NASA-TLX was used. For evaluation, the raw NASA-TLX was used which is an average workload score between 1 and 100 calculated by multiplying each rating by 5. Additionally, an overall workload score was calculated averaging the ratings on the six subscales (Said et al., 2020). A NASA-TLX score of 55/100 was expected which is based on the average score for the performance of cognitive tasks, physical activity and video gaming (Grier, 2015). Furthermore, after each training session, participants were asked to rate their perceived enjoyment level on a 5-point Likert scale.

#### Questionnaire Regarding Usability and Safety

A questionnaire was used to assess user experience and safety aspects. The questionnaire included a total of 19 items to assess each participants’ subjective perception of the exergame training sessions. Thirteen items were rated on a 5-point Likert scale ranging from 1 to 5 and assessed fun, motivation, excitement and diversification of the games, perceived improvements of motor coordination, perceived improvements of cognitive performance, intention to recommend this type of training to everyone as well as specifically to people with coordinative impairments, or to people with cognitive impairment, frequency of the training sessions, duration of the training sessions, feeling of safety during training, and fear of falling during training. In six further open questions the participants were asked for their favorite game, their least favorite game, their most challenging game, their least challenging game, what kind of positive effects resulting from the training were perceived, and general impressions of the training.

### Secondary Outcomes

The effects of the exergame intervention on physical and cognitive functions were examined as secondary outcome to receive first indications whether a full RCT of the intervention will be worthwhile and to determine whether there is a need for further development of the intervention (Abbott, 2014). For that purpose, several physical and cognitive tests were executed before and after the intervention or control period (T1- and T2-measurement).

#### Timed Up and Go Test

The Timed Up and Go (TUG) (Podsiadlo and Richardson, 1991) is a quickly executed test that only requires a chair and a stopwatch. It measures how much time participants need to perform the following task: Stand up from a chair, walk 3 m with comfortable speed, turn around, return to the chair and sit down again. The participants were asked to stand up without using their arms, if possible. However, if it was not possible, using their arms was allowed and noted. The test measured functional mobility and balance and can be used to detect change over time (Podsiadlo and Richardson, 1991).

#### Short Physical Performance Battery

The Short Physical Performance Battery (SPPB) developed by the National Institute on Aging (Short Physical Performance Battery (SPPB), 2021) is a tool (Mijnarends et al., 2013) for assessing motor functioning of the lower extremities. The test battery includes three physical tasks: Maintaining balance in different positions, standing up and sitting down five times as fast as possible and walking at comfortable speed. The total points achieved in all tests together as well as the completion time for the five times sit-to-stand subtask were used for further analyses.

#### Gait Performance

Normal walking speed was measured during single and dual task conditions while maximal walking speed was measured only during single task condition. In all tasks, participants had to walk along a straight walkway of 14 m. The time was measured during the 10 m in the middle providing the participants with 2 m for acceleration and deceleration, respectively. The 14- and the 10-m zones were marked with taped lines and the time was measured with a stopwatch as soon as the toes of the participant crossed the starting or the finishing line of the 10-m zone, respectively. For each task, the test was repeated twice and the average time was used for further analysis. If required, the use of assistive devices was allowed and documented by the local investigator. For the dual task condition participants had to count backward from 250 (first walk) and 245 (second walk) in steps of 7 (or 3, in case counting in steps of 7 was too difficult) during walking. Both are often used cognitive tasks in dual task paradigms (Bayot et al., 2020).

#### Step Reaction Time Test

The Step Reaction Time test (SRTT) was performed on the Dividat Senso. It measures psychomotor speed in terms of reaction to a visual stimulus using the lower extremities. On the screen, six gray triangles are depicted. As soon as one of these triangles turns black, participants have to react by stepping as quickly as possible in one of the six possible directions in which the stimulus appeared (right, front left, right, left, back right and back left). Average reaction time was used for further analyses.

#### Go/No-Go Test

The Go/No-Go Test was also performed on the Dividat Senso and measures selective attention and inhibition. Participants have to focus on a small gray dot in the middle of the screen. In a randomized order, crosses (+) and Xs (x) appear on the right and left side of the gray dot. Participants are asked to ignore the crosses and only react to the Xs by stepping as quickly as possible in the right direction. Average reaction time was used for further analyses.

#### Trail Making Test

The Trail Making Test (TMT) is a paper-pencil test consisting of two parts. Part A (TMT-A) mainly assesses processing speed (Crowe, 1998). Circled numbers from 1 to 25 are allocated randomly on a sheet which participants have to connect in the right order. Part B (TMT-B) mainly assesses mental flexibility (Crowe, 1998). Here, circled numbers and letters are randomly allocated on a sheet and the participants have to connect circled numbers and letters in the right order and in alternating manner. The required time to complete each task was measured in both parts (Tombaugh, 2004).

#### Color-Word Interference Test (D-KEFS)

The Color-Word Interference Test is a version of the Stroop Test (Stroop, 1935) and was developed as a part of the Delis–Kaplan Executive Function System, which is a battery of neuropsychological tests (Delis et al., 2001). The Color-Word Interference Test is an instrument to assess inhibition, mental flexibility and shifting consisting of four trials (Homack et al., 2005; Shunk et al., 2007; Jones Chesters, 2008). First is the *color naming trial* in which a sheet containing differently colored squares (red, green, blue) is presented to the participant who has to name the colors as quickly as possible. In the second *word reading trial*, the participant is presented with a sheet containing the words “red,” “green” and “blue” printed in black ink which the participant has to read aloud as quickly as possible. Third, the *inhibition trial* follows which is based on the Stroop test (Stroop, 1935). During this trial, the sheet presented to the participant contains the words “red,” “green” and “blue” printed incongruently in red, green or blue ink. The participant is asked to name the color of each word as quickly as possible while inhibiting reading the words. In the final *inhibition/switching trial*, the sheet looks the same as in the inhibition trial but additionally, half of the words are enclosed within boxes. The task is the same as in the inhibition trial expect for the enclosed words: The participant has to name the color of the non-enclosed words but read the word of the enclosed words. The time required to perform each trial was measured in each trial and errors counted in the third and fourth trial.

### Statistical Analysis

Descriptive statistics were used for the primary outcomes. For the statistical analysis of the secondary outcomes, R Statistics software (RStudio, Boston, MA, United States, version 3.6.3) was used. Data was first tested for normal distribution using the Shapiro–Wilk test and QQ plots. Afterward, the data were tested for homogeneity of variance using Levene’s test. A two-way mixed ANOVA was used to analyze intragroup differences between pre- and post-measurements, intergroup differences between the intervention and the control group and moreover, the interaction of the factors group and time. The interaction effect provides information whether the group assignment had an influence on the performance difference between pre- and post-measurements. Significance level was set at α = 0.05. If the two-way mixed ANOVA reported a significant group, time or interaction effect, data was further analyzed using *post hoc* tests. To calculate effect sizes of intragroup differences between post- and baseline measurements, a dependent *T*-test or its non-parametric equivalent (Wilcoxon signed rank test) was used. The effect size was interpreted using benchmarks describing the effect size as small (*r* ≥ 0.01), medium (*r* ≥ 0.3), or large (*r* ≥ 0.5) (Tomczak and Tomczak, 2014). In general, “per protocol analysis” was used which means that only participants with a sufficient adherence rate (≥70%) were included in the analysis of the effects.

## Results

### Demographics and Patient Flow

**FIGURE 1:**
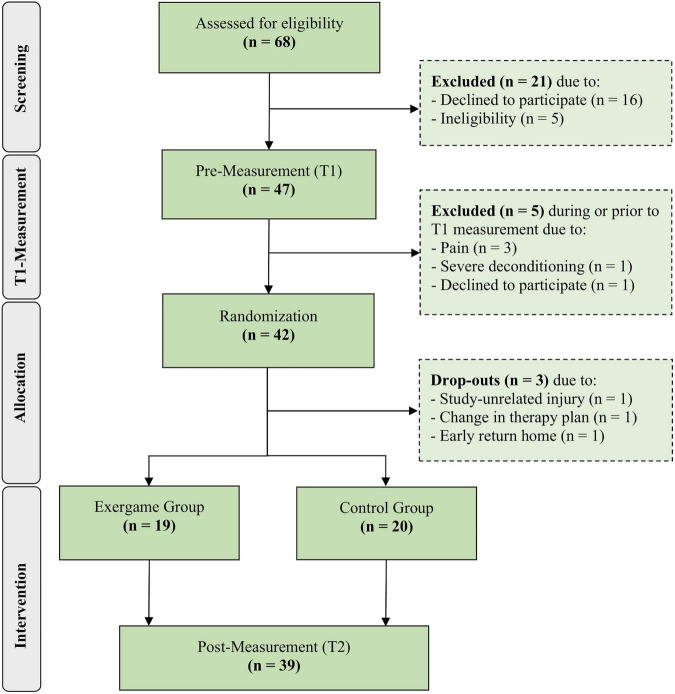
Flow diagram of screening and group allocation (based on Eldridge et al., 2016).

The demographic data are depicted in Table 1. There was no statistically significant difference in age, MMSE score, BMI, time between pre- and post-measurement, years of education or physical activity between the two groups. A total of 39 patients were included in the final analysis (Figure 1). The intervention period lasted between 8 and 23 days and the average amount of training sessions was 9.6.

**TABLE 1:** Demographics of study participants.

Variables	Exergame group	Control group	*P*-values
Number of participants	19	20	
Sex, Female:Male	[11:8]	[10:10]	
Age, years, mean (SD)	73.0 (8.8)	72.2 (9.8)	0.789
MMSE score, mean (SD)	27.3 (2.1)	28.0 (1.3)	0.208
BMI, kg/m2, median (IQR)	27.3 (5.3)	25.1 (5.3)	0.740
Reason for rehabilitation, %	(47) knee prosthesis (16) hip prosthesis (26) upper extremities (5) back (5) general rehabilitation	(30) knee prosthesis (45) hip prosthesis (15) upper extremities (5) back (5) general rehabilitation	
Time between pre- and post-measurement, days, median, (IQR)	15.0 (3.0)	12.0 (5.3)	0.075
Participants with fall history during last 12 months, %	26	45	
Years of education, years, mean (SD)	11.8 (2.4)	12.2 (3.0)	0.641
Regularly physically active, %	79	85	
Physically active, h/week, mean (SD)	4.3 (3.3)	6.3 (5.4)	0.165
Polypharmacy, %	79	60	
Comorbidities, %	(74) cardio-vascular diseases (68) internal / endocrine diseases (79) orthopedic diseases (5) neurological / psychiatric diseases (21) gastrointestinal diseases (5) eye/ear diseases (16) tumor	(65) cardio-vascular diseases (55) internal / endocrine diseases (95) orthopedic diseases (15) neurological / psychiatric diseases (20) gastrointestinal diseases (10) eye/ear diseases (10) tumor	

### Primary Outcomes

Clinicians were willing to recruit patients, and patients were willing to be randomized in either treatment arm. Within the clinical setting of orthopedic rehabilitation, the exergame intervention could be delivered as intended.

**FIGURE 2:**
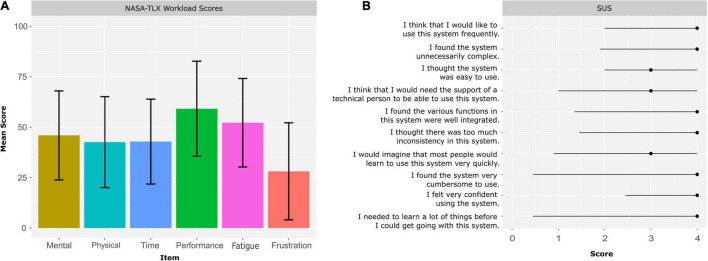
Feasibility outcomes. (A) Mean ratings ± standard deviation of each item of the NASA-TLX to assess training workload, (B) median and interquartile range of the ratings of each SUS item.

More than 69% of the 68 screened patients agreed to participate and 62% of the 68 screened patients were eventually included into the study and allocated to a study group (Figure 1). The attrition rate was 7% (*n* = 3 participants) and the dropout reasons were all study-unrelated (Figure 1). Two participants had to quit prior to the first training and one participant left the clinic after one training due to personal reasons. Consequently, no dropouts occurred for intervention-related reasons and, therefore, the intervention-related attrition rate was 0%. The average adherence rate was 99% and the reasons for non-adherence were acute back-pain and acute stomach-ache. No adverse events occurred during the training sessions (and also not during the pre- and post-assessments). The mean rating of the enjoyment level perceived by the participants in all training session was 4.78 (SD = 0.52) on a 5-point Likert scale. The average scores of each item of the raw NASA-TLX are depicted in Figure 2A and the average overall raw NASA-TLX score was 45.5 (SD: 10.40) on a scale from 0 to 100. The mean SUS score was 83.6 (SD: 13.72) on a scale from 0 to 100 and the ratings of each SUS item are depicted in Figure 2B. The results of the self-tailored questionnaire are summarized in Table 2.

**TABLE 2:** Self-tailored Questionnaire regarding usability and safety.

The training on the Dividat Senso was fun.	Completely true (14)	Quite true (5)	More or less true (0)	Rather untrue (0)	Completely untrue (0)
I was motivated for the training.	Completely true (12)	Quite true (6)	More or less true (1)	Rather untrue (0)	Completely untrue (0)
I found the games exciting.	Completely true (12)	Quite true (7)	More or less true (0)	Rather untrue (0)	Completely untrue (0)
I found the games diversified.	Completely true (17)	Quite true (2)	More or less true (0)	Rather untrue (0)	Completely untrue (0)
I think that the training on the Dividat Senso helped to improve my coordination (e.g., balance, reaction).	Completely true (11)	Quite true (6)	More or less true (2)	Rather untrue (0)	Completely untrue (0)
I think that the Training on the Dividat Senso helped to improve my cognitive functions (e.g., memory, concentration).	Completely true (10)	Quite true (7)	More or less true (2)	Rather untrue (0)	Completely untrue (0)
I would recommend the training on the Dividat Senso to people with coordinative or balance impairments.	Completely true (16)	Quite true (3)	More or less true (0)	Rather untrue (0)	Completely untrue (0)
I would recommend the training on the Dividat Senso to people with cognitive impairments.	Completely true (13)	Quite true (2)	More or less true (4)	Rather untrue (0)	Completely untrue (0)
I would recommend the training on the Dividat Senso to other people in general.	Completely true (15)	Quite true (3)	More or less true (1)	Rather untrue (0)	Completely untrue (0)
How would you rate the frequency of the training sessions (5x per week)?	Too low (0)	Rather low (2)	Optimal (14)	Rather high (2)	Too high (1)
How would you rate the duration of the training sessions (approx. 10–15 min)?	Too short (0)	Rather short (3)	Optimal (16)	Rather long (0)	Too long (0)
How safe did you feel during the training sessions?	Very unsafe (0)	Rather unsafe (2)	Not safe nor unsafe (1)	Rather safe (9)	Very safe (7)
Were you afraid of falling during the training sessions?	Never (15)	Hardly ever (2)	Sometimes (2)	Often (0)	Always (0)
Which game did you like the most?	Targets (7), Habitats (6), Simple (2), Ski (2), Hexagon (1), All (1), other games (0)				
Which game did you like the least?	Ski (5), Flexi (3), Hexagon (2), Snake (1), Habitats (1), Simple (1), Simon (1), Targets (1), None (4), other games (0)				
Which game was the most challenging?	Hexagon (5), Flexi (5), Ski (5), Habitats (3), Targets (1), other games (0)				
Which game was the least challenging?	Simple (15), Habitats (3), Flexi (1), other games (0)				
Have you noticed any positive effects (physical, psychological, cognitive) during the training period?	Yes (18), No (1)				
If yes, which positive effects have you noticed?	Reaction (5), attention (3), stability (3), concentration (2), balance (2), coordination (2), psychological well-being (2), cognitive (2), feeling safer (2)				

*Numbers in brackets represent absolute values of the frequencies in which each answer was given.*

### Secondary Outcomes

**FIGURE 3:**
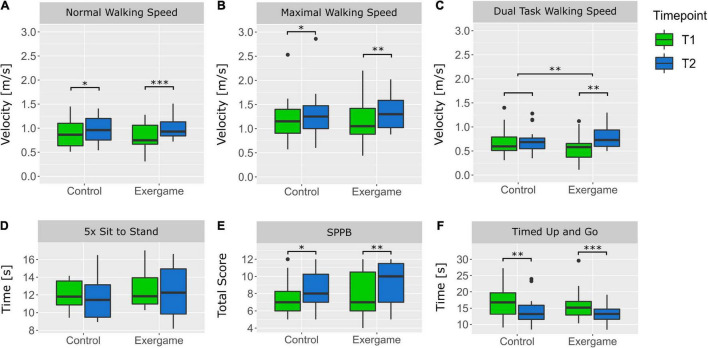
Boxplots of physical outcomes of each group at pre- and post-measurements. **(A)** Velocity for normal walking 10 m, **(B)** maximal velocity for walking 10 m, **(C)** velocity for walking 10 m while dual tasking, **(D)** Time required to stand up five times, **(E)** Total Score achieved in the Short Physical Performance Battery (SPPB), **(F)** Time required for the Timed Up and Go (TUG). *P < 0.05, ^**^P < 0.01, ^***^P < 0.001.

**FIGURE 4:**
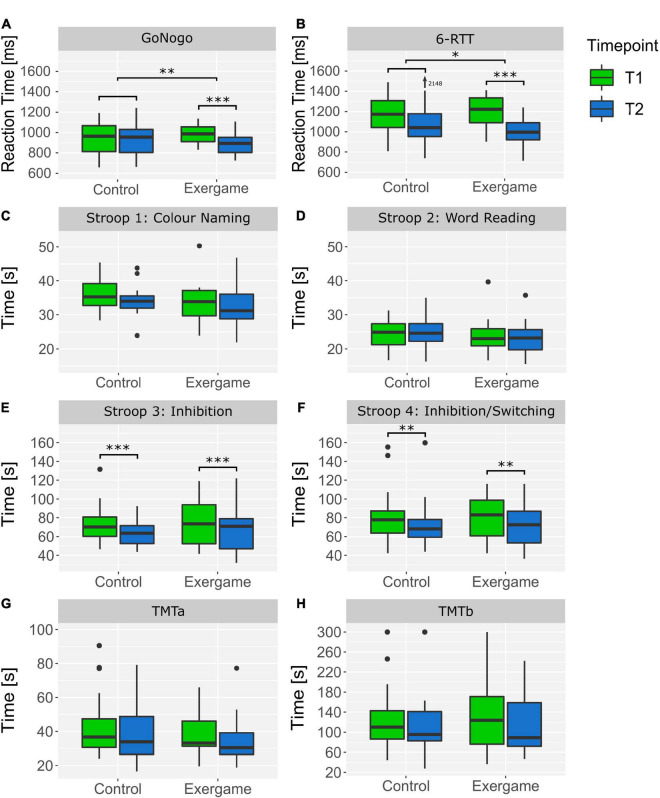
Boxplots of cognitive outcomes of each group at pre- and post-measurements. **(A)** Reaction Time in the Go/No-Go Test on the Dividat Senso, **(B)** Reaction Time in the 6-Step Reaction Time test (SRTT) on the Dividat Senso, **(C)** Time required for the first Stroop task (Color Naming), **(D)** time required for the second Stroop task (Word Reading), **(E)** time required for the third Stroop task (Inhibition), **(F)** time required for the fourth Stroop task (Inhibition/Switching), **(G)** time required for the Trail Making Test (TMT) part A, **(H)** time required for the Trail Making Test (TMT) part B. *P < 0.05, ^**^P < 0.01, ^***^P < 0.001.

The Shapiro–Wilk test reported a significant non-normal distribution of the data in all outcome measures expect Stroop1 time, five times sit to stand (FTSST) and Go/No-Go average reaction time, which was confirmed by QQ plots examination. The Levene’s test reported non-significant differences between groups and time-points of each outcome and therefore homogeneous variances can be assumed. The results of the robust two-way mixed ANOVA and the corresponding effect sizes are depicted in Table 3. *Post hoc* tests reported a significant time effect for normal walking speed (Ψ = −0.13, *P* < 0.001^***^), maximal walking speed (Ψ = −0.15, *P* < 0.001^***^), Stroop3 errors corrected (Ψ = 0.92, *P* = 0.014*), Stroop3 time (Ψ = 7.67, *P* < 0.001^***^), Stroop4 time (Ψ = 7.22, *P* < 0.001^***^), SPPB total score (Ψ = −0.82, *P* = 0.004^**^), TUG time (Ψ = 2.24, *P* < 0.001^***^), Go/No-Go average reaction time (Ψ = 53.61, *P* = 0.002^**^) and SRTT average reaction time (Ψ = 166.8, *P* < 0.001^***^) and a non-significant time effect for dual task walking speed (Ψ = 0.08, *P* = 0.088) and Stroop 1 time (1.18, *P* = 0.178). Furthermore, *post hoc* tests reported a significant interaction effect for dual task walking speed (Ψ = 0.23, *P* = 0.002^**^), Go/No-Go average reaction time Ψ = (−81.16, *P* = 0.008^**^) and SRTT average reaction time (Ψ = −134.60, *P* = 0.022*). In Figure 3, the boxplots for the physical and dual-task outcomes and in Figure 4, the boxplots for the cognitive outcomes are depicted. Highest performance improvements between pre- and post-measurements were found in the exergame group in the outcome measures SRTT, Go/No-Go and dual task walking speed. The effect sizes for change over time in each group are summarized in Table 2.

**TABLE 3:** Results of each outcome measure across groups and timepoints.

Outcome measures	Exergame group (EG)			Control group (CG)			Q-value (df), *P*-value, Effect size (η ^2^)		
	T1	T2	*N*	T1	T2	*N*	T1-T2	EG-CG	Interaction
Normal Walking Speed	0.75 (0.40)	0.93 (0.29)	19	0.87 (0.47)	0.96 (0.45)	20	Q(1,21.74) = 16.48 *P* < 0.001***, η^2^ = 0.43	Q(1,21.29) = 0.08 *P* = 0.776, η^2^ = 0.00	Q(1,21.74) = 0.32 *P* = 0.577, η^2^ = 0.01
Maximal Walking Speed	1.05 (0.54)	1.3 (0.57)	19	1.15 (0.50)	1.25 (0.48)	19	Q(1,22.26) = 14.93 *P* < 0.001***, η^2^ = 0.40	Q(1,23.38) = 0.04 *P* = 0.846, η^2^ = 0.00	Q(1,22.26) = 0.62 *P* = 0.441, η^2^ = 0.03
Dual-Task Walking Speed	0.58 (0.28)	0.73 (0.34)	18	0.60 (0.28)	0.69 (0.22)	20	Q(1,17.17) = 10.65 *P* = 0.005**. η^2^ = 0.38	Q(1,20.01) = 0.01 *P* = 0.930, η^2^ = 0.00	Q(1,17.17) = 5.25 *P* = 0.035*, η^2^ = 0.23
TMTA	33.10 (14.77)	30.40 (12.67)	19	36.63 (16.79)	33.85 (22.22)	20	Q(1,19.02) = 4.34 *P* = 0.051, η^2^ = 0.19	Q(1,19.21) = 0.66 *P* = 0.428, η^2^ = 0.03	Q(1,19.02) = 0.34 *P* = 0.565, η^2^ = 0.02
TMTB	123.72 (95.18)	88.75 (86.88)	19	110.19 (56.52)	95.25 (58.47)	18	Q(1,23.23) = 2.98 *P* = 0.098, η^2^ = 0.11	Q(1,23.84) = 0.09 *P* = 0.767, η^2^ = 0.00	Q(1,23.23) = 0.05 *P* = 0.646, η^2^ = 0.00
Stroop1 Time	33.87 (7.45)	31.15 (7.25)	19	35.27 (6.40)	34 (3.57)	20	Q(1,19.78) = 5.14 *P* = 0.035*, η^2^ = 0.21	Q(1,19.82) = 1.27 *P* = 0.273, η^2^ = 0.06	Q(1,19.78) = 0.05 *P* = 0.822, η^2^ = 0.00
Stroop2 Time	23.00 (4.99)	23.19 (5.89)	19	24.88 (6.12)	24.58 (5.19)	20	Q(1,20.02) = 0.03 *P* = 0.872, η^2^ = 0.00	Q(1,22.00) = 1.83 *P* = 0.190, η^2^ = 0.08	Q(1,20.02) = 0.03 *P* = 0.860, η^2^ = 0.00
Stroop3 Time	73.5 (41.61)	70.93 (32.02)	19	70.24 (20.51)	63.60 (19.17)	20	Q(1,20.71) = 24.23 *P* < 0.001***, η^2^ = 0.54	Q(1,17.23) = 0.19 *P* = 0.669, η^2^ = 0.01	Q(1,20.71) = 0.02 *P* = 0.878, η^2^ = 0.00
Stroop3 Errors Not Corrected	0.00 (2.00)	0.00 (2.00)	19	0.00 (0.25)	0.00 (1.00)	20	Q(1,15.49) = 0.18 *P* = 0.680, η^2^ = 0.01	Q(1,15.18) = 1.67 *P* = 0.216, η^2^ = 0.1	Q(1,15.49) = 0.02 *P* = 0.879, η^2^ = 0.00
Stroop3 Errors Corrected	2.00 (1.50)	0.00 (2.00)	19	2.00 (3.25)	1.00 (1.25)	20	Q(1,16.59) = 12.90 *P* = 0.002**, η^2^ = 0.44	Q(1,21.69) = 0.00 *P* = 0.995, η^2^ = 0.00	Q(1,16.59) = 0.19 *P* = 0.667, η^2^ = 0.01
Stroop4 Time	83.00 (37.92)	72.5 (33.7)	19	77.78 (23.37)	68.21 (18.90)	20	Q(1,21.16) = 38.43 *P* < 0.001***, η^2^ = 0.64	Q(1,19.70) = 0.15 *P* = 0.699, η^2^ = 0.00	Q(1,21.16) = 0.13 *P* = 0.730, η^2^ = 0.00
Stroop4 Errors Not Corrected	2.00 (3.50)	1.00 (2.00)	19	1.00 (3.00)	1.00 (2.00)	20	Q(1,18.69) = 1.27 *P* = 0.274, η^2^ = 0.06	Q(1,17.53) = 0.61 *P* = 0.445, η^2^ = 0.03	Q(1,18.69) = 0.48 *P* = 0.499, η^2^ = 0.03
Stroop4 Errors Corrected	1.00 (1.50)	1.00 (2.00)	19	1.00 (2.25)	1.00 (2.25)	20	Q(1,21.68) = 0.01 *P* = 0.908, η^2^ = 0.00	Q(1,19.54) = 0.00 *P* = 0.964, η^2^ = 0.00	Q(1,21.68) = 0.15 *P* = 0.698, η^2^ = 0.00
5TSTS	11.84 (2.97)	12.25 (5.10)	9	11.80 (2.71)	11.43 (3.68)	12	Q(1,7.65) = 0.27 *P* = 0.621, η^2^ = 0.03	Q(1,7.56) = 0.32 *P* = 0.589, η^2^ = 0.04	Q(1,7.56) = 0.02 *P* = 0.882, η^2^ = 0.00
SPPB total score	7.00 (4.50)	10.00 (4.50)	19	7.00 (2.25)	8.00 (3.25)	20	Q(1,21.92) = 15.63 *P* < 0.001***, η^2^ = 0.42	Q(1,19.99) = 1.07 *P* = 0.313, η^2^ = 0.06	Q(1,21.92) = 0.07 *P* = 0.794, η^2^ = 0.00
TUG	15.16 (4.19)	13.21 (3.16)	19	16.78 (6.54)	13.19 (4.36)	19	Q(1,23.67) = 26.28 *P* < 0.001***, η^2^ = 0.53	Q(1,23.96) = 0.45 *P* = 0.508, η^2^ = 0.02	Q(1,23.67) = 1.01 *P* = 0.325, η^2^ = 0.04
Go/No-Go average Reaction Time	986.83 (144.08)	894.24 (149.28)	19	964.04 (254.37)	955.30 (226.55)	19	Q(1,23.38) = 16.77 *P* < 0.001***, η^2^ = 0.42	Q(1,19.54) = 0.02 *P* = 0.879, η^2^ = 0.00	Q(1,23.38) = 6.71 *P* = 0.016*, η^2^ = 0.22
SRTT average Reaction Time	1,223.1 (245.53)	996.2 (167.32)	19	1,173.55 (265.61)	1,078.21 (245.56)	20	Q(1,20.16) = 27.73 *P* < 0.001***, η^2^ = 0.58	Q(1,20.83) = 0.13 *P* = 0.725, η^2^ = 0.00	Q(1,20.16) = 5.23 *P* = 0.033*, η^2^ = 0.21

Data presented as median (IQR, Interquartile range); TMT, Trail Making Test, Stroop1, Color naming trial; Stroop2, Word reading trial; Stroop3, Inhibition trial; Stroop4, Inhibition/Switching trial; 5TSTS, 5 times standing up from a chair (part of SPPB); SPPB, Short Physical Performance Battery; TUG, Timed Up and Go; SRTT, Step Reaction Time Test. *p < 0.05, **p < 0.01, ***p < 0.001.

## Discussion

### Feasibility

The main aim of this study was to test the feasibility of an exergame intervention in orthopedic inpatient rehabilitation of geriatric patients. All outcome measures regarding usability, safety and acceptability suggest high feasibility and therefore this is an indication that a full RCT of the intervention is worthwhile. There seems to be, furthermore, no pressing need for further development of the intervention before such a RCT can be started for geriatric inpatients. The adherence rate of 99% is very high and goes in line with previous studies examining the adherence to technology-based exercise interventions in older people as reviewed by Valenzuela et al. (2018). However, in the present study and in most studies in that systematic review, the exergame training was supervised closely. It is possible, that the therapeutic alliance contributed to the high adherence rate (Moore et al., 2020). In the present study, many participants emphasized that they appreciated the close supervision by the same person which is not usual in a rehabilitation setting (i.e., exergame training was always supervised by the same person (the study investigator), whereas the rest of the treatments were conducted by different/changing therapists). Similarly, a very low attrition rate was observed, and no dropout occurred due to intervention-related reasons. This results in an intervention-related attrition rate of 0% which has also been observed in several previous studies in older people (Williams et al., 2010; De Bruin et al., 2011; Toulotte et al., 2012; Kim et al., 2013; Lai et al., 2013; Silveira et al., 2013; Lee et al., 2014; Chao et al., 2015). In addition, both, the high adherence and low attrition rate may be further explained by the high motivational potential of exergames (Proffitt et al., 2015; Valenzuela et al., 2018). Many older people enjoy playing exergames (Williams et al., 2010; Franco et al., 2012; Jorgensen et al., 2013) which was also the case in this study in which high motivation and enjoyment levels were perceived during the training sessions. Furthermore, the participants generally felt safe during training and only very few were concerned about falling. The absence of any adverse events confirms this high sense of security and suggests that exergaming is a safe training intervention not only in healthy older people (Valenzuela et al., 2018), but also in more frail people undergoing rehabilitation. However, the applied training load might have been rather low. The raw NASA-TLX score of 45.5 was below the expected score of 55. The score of 45.5 is comparable to executing cognitive tasks (mean score: 46.0), but lower than physical activities (mean score: 62.0) and video gaming (56.5) as summarized in a meta-analysis by Grier (2015). Therefore, it seems that the physical component of the training was perceived as easier compared to other physical activities while the cognitive workload was comparable to other cognitive tasks. In addition, the perceived workload score tends to be higher in other video games, which could mean that overall workload of the conducted training might have been rather low. Nevertheless, most participants rated the frequency and duration of the training sessions as optimal. This could mean that the workload in terms of number of training sessions per week and duration of training sessions were sufficient, but the training intensity tended to be low. The SUS score of 83.6 can be described as good (Bangor et al., 2009) and suggests that the participants generally had a positive user experience and no major problems in their interaction with the training device were perceived. However, the item-specific SUS ratings suggest that technical support is required for a successful use of the exergame system. Since the Dividat Senso was created for clinical purposes mostly under supervision, the supervisor is indirectly part of the training system. Consequently, the training supervisor should be familiar with the Dividat Senso to ensure fast problem-solving of potential technical problems. In general, the participants were very satisfied with the exergame intervention. Almost all participants subjectively noticed positive effects on physical, cognitive, or psychological aspects during the intervention period and would recommend the exergame training to other people. Moreover, twice as many participants of the intervention group were willing to prolong the stay at the rehabilitation clinic compared to the control group. This might be because some of the participants saw the benefits of the additional exergame training and wanted to profit more from this opportunity. To summarize, in this study, it was shown for the first time that exergaming on the Dividat Senso in geriatric patients is feasible in terms of usability, safety, and acceptability. Thus, exergaming can be successfully incorporated in the rehabilitation program of geriatric patients in inpatient rehabilitation clinics.

### Physical and Cognitive Functioning

We hypothesized that the program with additional exergaming would be more meaningful for patients when compared to the traditional rehabilitation program. In all physical and cognitive outcome measures, the exergame group made equal or higher performance improvements compared to the control group. Both groups significantly improved in most physical and cognitive outcome measures. The exergame group also significantly improved in Go/No-Go and SRTT reaction time, and dual task walking speed. Effect sizes ranged between 0.25 and 0.85 and mostly favor the exergame training group (Table 4). A significant interaction effect between group and time reveals that the group allocation had a significant influence on the performance in these tests. In the intervention group, stepping capacity could therefore be improved in terms of a reduced choice reaction time which was also shown by Crotty et al. (2011). In addition, the intervention group was able to increase their walking speed while executing a cognitive task. Furthermore, this group showed walking speed change well beyond the 0.13 m per second Minimal Detectable Change Values (MDC) that can be expected in short term rehabilitation (Middleton et al., 2015) whereas the values for the control group remained within the MDC. This result is consistent with previous findings describing the combination of physical-cognitive training to have superior effects on dual task walking speed than physical training alone (Tait et al., 2017; Raichlen et al., 2020). It can, thus, be concluded that the exergame intervention in this study had a superior effect on the physical-cognitive tasks compared to the rehabilitation program alone. Furthermore, the effect sizes of most outcome measures are higher in the exergame training group compared to the control group (Table 4). However, no significant differences were found between the groups regarding the outcome measures assessing either physical or cognitive functions alone. This is in contrast to previous studies which reported significant effects of exergaming on cognitive functions (Stanmore et al., 2017), functional mobility or balance (Crotty et al., 2011; Jorgensen et al., 2013; Jung et al., 2015; Park et al., 2015; Padala et al., 2017). However, in most of these studies, the intervention period lasted for at least 8 weeks and for balance assessment, the Berg Balance Scale (BBS) was used. Moreover, in some studies (Jorgensen et al., 2013; Schoene et al., 2013), a passive control group was used. These methodological differences might explain the absence of significant differences between the groups in the individual cognitive and physical outcomes in this study. On the one hand, it is possible that the exergame training was cognitively and/or physically not sufficiently demanding to achieve the optimal training stimulus to induce plastic alterations measurable by the outcome measures (Lövdén et al., 2010). On the other hand, a previously reported dose-response effect between cognitive-motor training and cognitive benefits suggests that the benefits of the applied exergame training could increase and become measurable by increasing training dosage (Bamidis et al., 2015). Thanks to the gamification of exercise, exergaming has the potential to increase patients’ motivation and long-term adherence to exercise routines (Proffitt et al., 2015; Kappen et al., 2019; Randriambelonoro et al., 2020), that stretch beyond their stay in the inpatient clinic. Exergaming can make a rehabilitation program more entertaining and therefore increase the success of the entire rehabilitation program (Bonnechère et al., 2016). Our data warrant performance of a RCT that is performed over a longer (outpatient) rehabilitation period and that should assess effectiveness. Furthermore, it was shown that exergaming has a significant effect on stepping capacity and walking during dual task conditions. Since both are known fall risk factors (Muir-Hunter and Wittwer, 2016; Okubo et al., 2017, 2021; Bayot et al., 2020), exergaming on the Dividat Senso could be a beneficial supplement to conventional rehabilitation therapies to reduce fall risk of geriatric patients.

**TABLE 4:** Effect sizes representing change over time in each group.

Outcome measures	Exergame group (EG)	Control group (CG)
	T1-T2	T1-T2
Normal Walking Speed	*r* = 0.76, *P* < 0.001***	*r* = 0.48, *P* = 0.032*
Maximal Walking Speed	*r* = 0.66, *P* = 0.004**	*r* = 0.46, *P* = 0.044*
Dual-Task Walking Speed	*r* = 0.77, *P* = 0.001**	*r* = 0.25, *P* = 0.263
TMTA	*r* = 0.42, *P* = 0.073	*r* = 0.43, *P* = 0.053
TMTB	*r* = 0.34, *P* = 0.137	*r* = 0.33, *P* = 0.157
Stroop1 Time	*r* = 0.35, *P* = 0.131	*r* = 0.43, *P* = 0.143
Stroop2 Time	*r* = 0.40, *P* = 0.083	*r* = 0.23, *P* = 0.314
Stroop3 Time	*r* = 0.71, *P* < 0.001***	*r* = 0.74, *P* < 0.001***
Stroop3 Errors Not Corrected	*r* = 0.12, *P* = 0.447	*r* = 0.15, *P* = 0.504
Stroop3 Errors Corrected	*r* = 0.64, *P* = 0.006**	*r* = 0.48, *P* = 0.025*
Stroop4 Time	*r* = 0.70, *P* = 0.001**	*r* = 0.59, *P* = 0.006**
Stroop4 Errors Not Corrected	*r* = 0.02, *P* = 0.97	*r* = 0.24, *P* = 0.235
Stroop4 Errors Corrected	*r* = 0.20, *P* = 0.592	*r* = 0.09, *P* = 0.645
5TSTS	*r* = 0.22, *P* = 0.515	*r* = 0.56, *P* = 0.173
SPPB total score	*r* = 0.76, *P* = 0.001**	*r* = 0.40, *P* = 0.036*
TUG	*r* = 0.77, *P* < 0.001***	*r* = 0.64, *P* = 0.005**
Go/No-Go average Reaction Time	*r* = 0.77, *P* < 0.001***	*r* = 0.27, *P* = 0.268
SRTT average Reaction Time	*r* = 0.85, *P* < 0.001***	*r* = 0.38, *P* = 0.092

Results of Wilcoxon signed rank test. r = effect size, calculated: $\text{r}=\frac{Z}{\sqrt{n}}$(Z, Absolute standardized test statistic; n, number of pairs); P, P-Value; TMT, Trail Making Test; Stroop1, Color naming trial; Stroop2, Word reading trial; Stroop3, Inhibition trial; Stroop4, Inhibition/Switching trial; 5TSTS, 5 times standing up from a chair (part of SPPB); SPPB, Short Physical Performance Battery; TUG, Timed Up and Go; SRTT, Step Reaction Time Test. *p < 0.05, **p < 0.01, ***p < 0.001.

### Study Limitations

In this study, the exergame intervention was examined in geriatric patients undergoing inpatient rehabilitation and the generalization to other population groups and settings is limited. Further studies are required to test the feasibility and effects of exergaming on the Dividat Senso in other patient groups undergoing inpatient rehabilitation but also in geriatric patients and other patient groups undergoing outpatient rehabilitation for longer durations. Another limitation is that all measurements and training sessions were conducted and supervised by the same local investigator. Consequently, blinding was only possible for the pre-measurements but not for group assignment and post-measurements. A further limitation is the short duration of the intervention. A longer intervention period is needed where effectiveness should be investigated. However, the standard prescription for rehabilitation in an orthopedic or geriatric rehabilitation clinic in Switzerland ranges between 2 and 3 weeks. Since this study aimed to test exergaming in a realistic rehabilitation setting it was important not to artificially extend the duration of the rehabilitation. Another point that should be critically discussed is the chosen outcome measures. No feasibility measures were assessed for the control group which received just the standard treatment; thus a direct comparison of e.g., the required resources is impossible. In addition to this, about half of the participants were not able to execute the Five Times Sit to Stand Test which is a part of the SPPB. Still, the SPPB total score revealed high performance differences between pre- and post-measurements in the intervention group above the substantial change estimates (Perera et al., 2006). However, the use of an easier sit to stand test such as the modified 30 Second Sit to Stand Test (m30STS) (McAllister and Palombaro, 2020) would provide additional information on functional mobility for a future RCT in this frail population group. A further limitation is that the dual task performance was only assessed by walking speed while information about the cognitive performance was not assessed. Therefore, no statement can be made if the improvements in the exergame group is a result of the task prioritization or improved task switching ability. Finally, despite the strategies for individual training adaptations described in the “Materials and Methods” section, it is possible that the training intensity was not sufficiently high to provide the optimal stimulus to each participant. The quantitative assessment of the patients’ subjective perception of game difficulty would be a further option to individually adapt training load.

### Conclusion and Outlook

In this pilot feasibility study, it was shown that exergaming using the Dividat Senso is a feasible, safe and effective intervention that can readily be integrated in the rehabilitation programs of geriatric inpatients. The high adherence rate, low attrition rate and high acceptability suggest that exergaming offers a great opportunity to make a rehabilitation program more entertaining and increase the motivation of the patients to adhere to their exercise routines while staying in a rehabilitation clinic. Moreover, exergaming on the Dividat Senso has the potential to improve stepping capacity and dual task walking speed in only a few weeks. Both are important fall risk factors and therefore exergaming could be beneficial in reducing fall risk in geriatric patients as previously indicated by a systematic review (Schoene et al., 2014). Furthermore, if the intervention period could be prolonged, more beneficial effects, also on single cognitive and physical functions, might be expected. Consequently, the continuation of the exergame training within the scope of outpatient rehabilitation or as a home-based approach after the end of the inpatient rehabilitation is warranted. The development of home-based exergame systems seems to offer potential for future fall-prevention strategies. Future studies using longer time frames should place a focus on adjusting the training load to each participants’ level and also assess dose-response effects while progressing through a rehabilitation program.

## Data Availability Statement

The raw data supporting the conclusions of this article will be made available by the authors, without undue reservation.

## Ethics Statement

This study’s design was reviewed and approved by the Cantonal Ethics Committee of Zurich, Switzerland (Reg. No. 98 2020-02388). All participants provided their written informed consent.

## Author Contributions

EG, MA, and EB designed the study. FG was responsible for the recruitment. FG, EG, and MA supervised the data collection process. PA collected and analyzed the data and drafted the first manuscript. All authors critically revised the manuscript and approved the final submitted version.

## Conflict of Interest

EB was a co-founder of Dividat, the spin-off company that developed the video step platform used for the training of the seniors and is associated to the company as an external advisor. No revenue was paid (or promised to be paid) directly to EB or his institution over the 36 months prior to submission of the work. MA works as a Head of Research at Dividat. The remaining authors declare that the research was conducted in the absence of any commercial or financial relationships that could be construed as a potential conflict of interest.

## Publisher’s Note

All claims expressed in this article are solely those of the authors and do not necessarily represent those of their affiliated organizations, or those of the publisher, the editors and the reviewers. Any product that may be evaluated in this article, or claim that may be made by its manufacturer, is not guaranteed or endorsed by the publisher.
