# Supplementary material for: Feasibility of Cognitive-Motor Exergames in Geriatric Inpatient Rehabilitation: A Pilot Randomized Controlled Study
Source: Front Aging Neurosci. 2021 Nov 29;13:739948. doi: 10.3389/fnagi.2021.739948 (PMC8667343; doi:10.3389/fnagi.2021.739948)
Supplement: Supplementary file 1 [file Table_1.DOCX]

Supplementary material

| **Game** | **Description** | **Main cognitive function(s)** |
| --- | --- | --- |
| **Simple**  **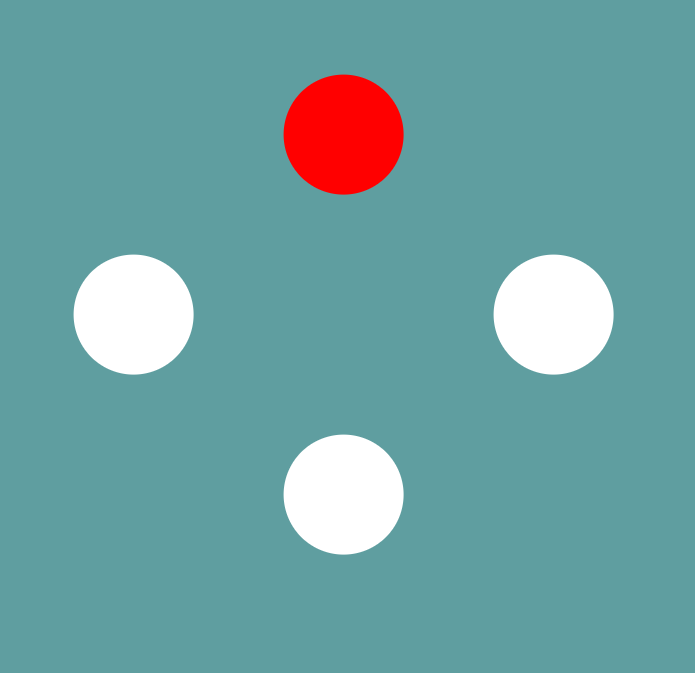** | Four white dots are depicted on the screen. As soon as one dot turns red, the participant has to step in the right direction as quickly as possible. | Focused attention  Psychomotor speed |
| **Divided**  **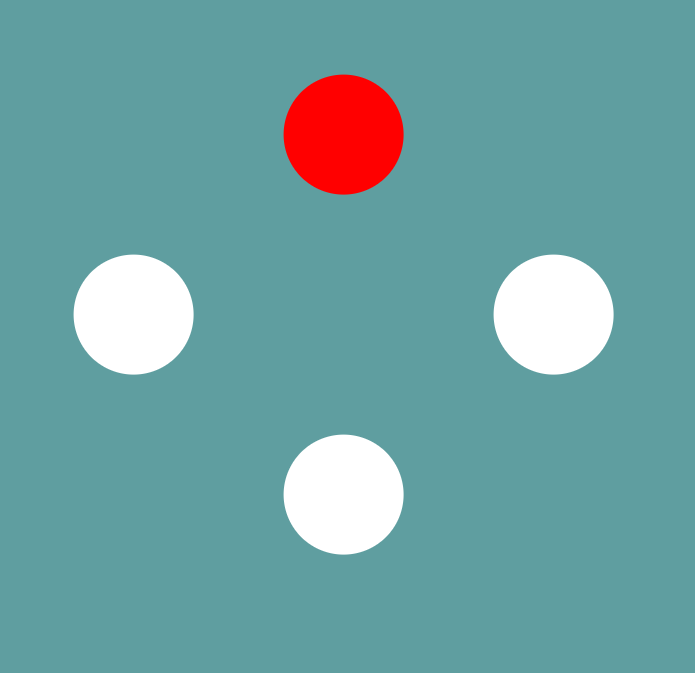** | Same as simple but with an additional acoustic signal: In between the appearance of a red dot, a high or low acoustic signal sometimes appears on which the participant has to react by stepping forward or backward, respectively. | Selective attention |
| **Birds**  **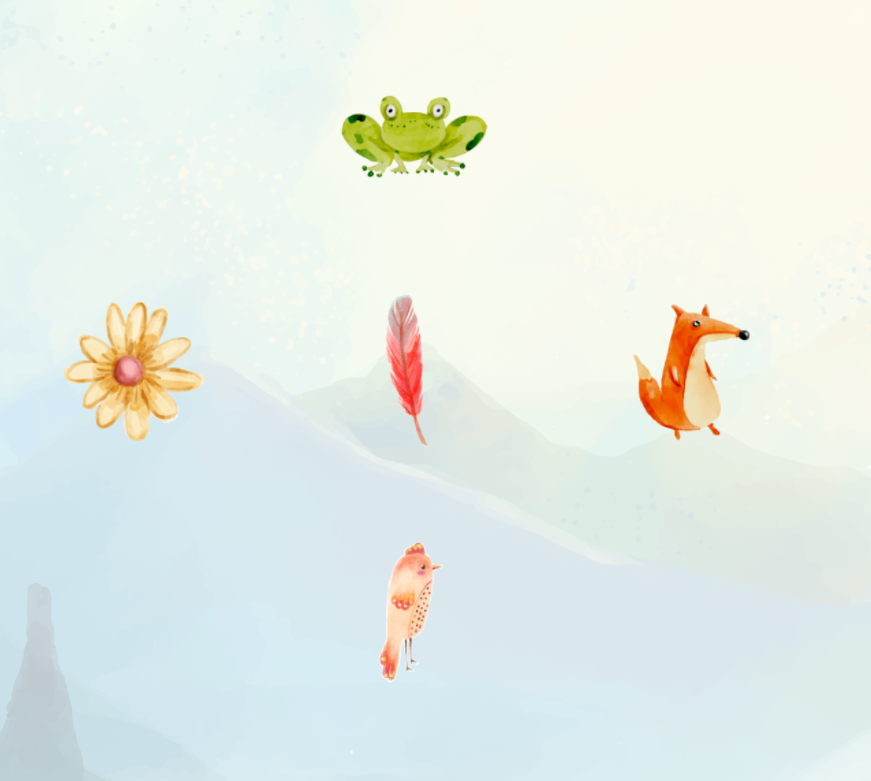** | A feather is displayed in the centre of the screen and is surrounded by 4 different items. The participant has to quickly step in the direction of the bird having the same colour as the feather. | Selective attention |
| **Targets**  **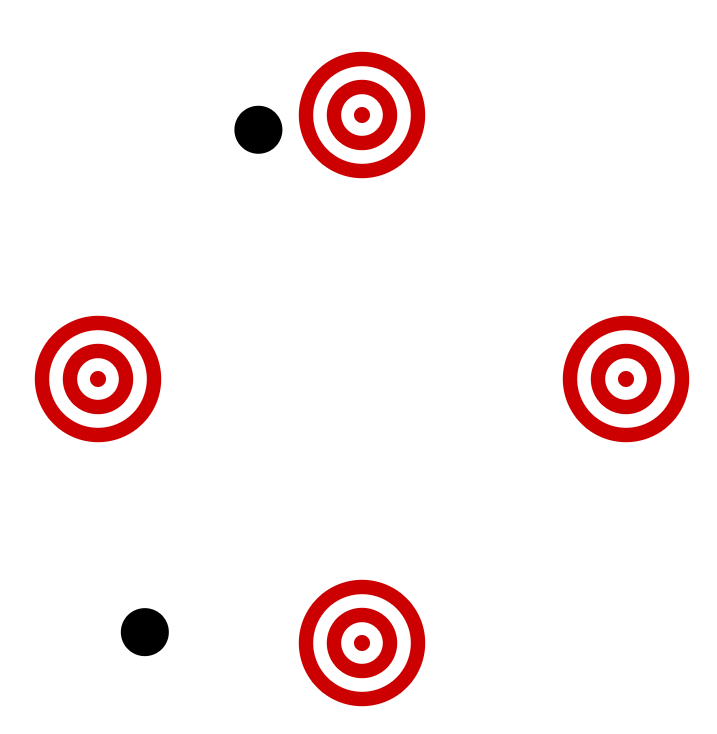** | Four targets are depicted on the screen. Black dots “fly” randomly across the screen. As soon as the black dot hits the center of a target, the participant has to step in the respective direction. | Foresighted thinking  Anticipation  Timing |
| **Habitats**  **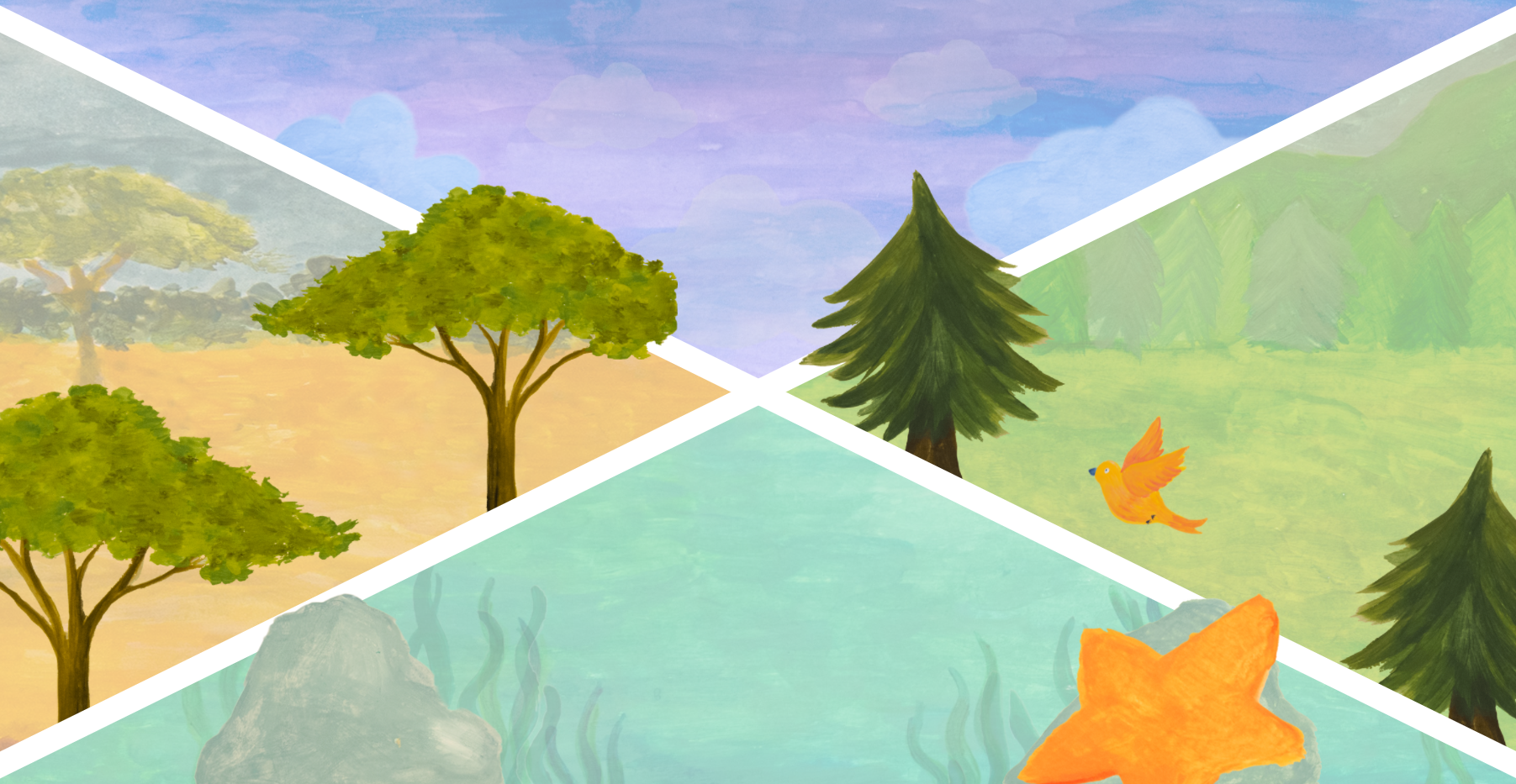** | Four habitats are displayed on the screen. Four different animals randomly appear in one habitat. The participant only has to step in the respective direction if the animal does not fit the habitat. | Selective attention  Inhibition |
| **Hexagon**  **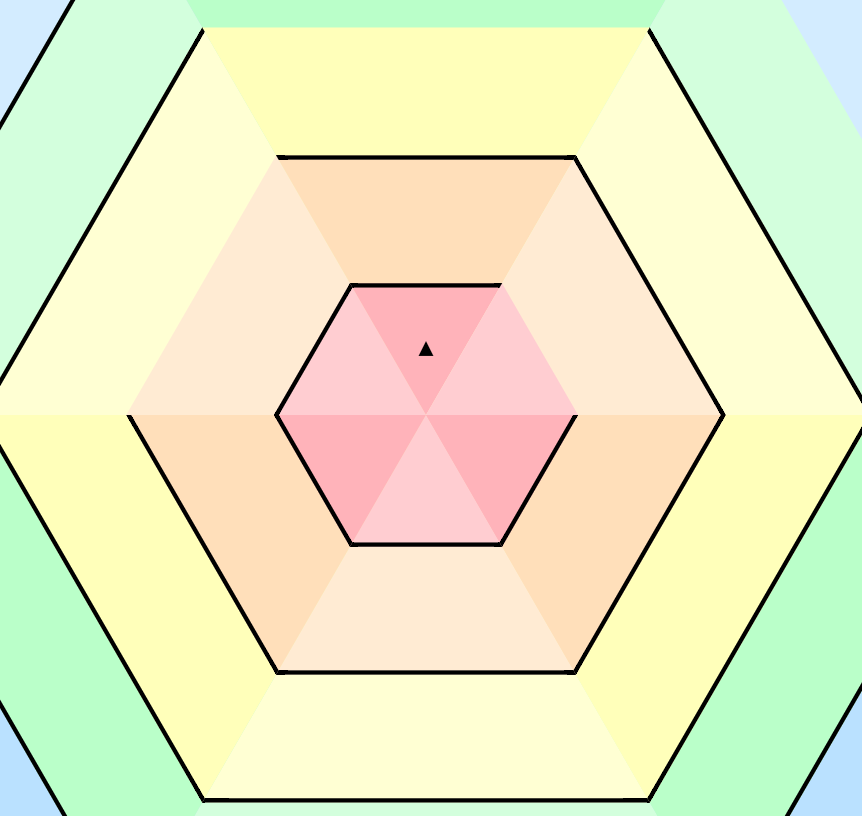** | An arrow is placed in the middle of many hexagons. The hexagons continuously decrease in size. The participant has to rotate the hexagon to the left or to the right by stepping to the respective direction, so that the open side is at the top and the arrow can leave the hexagon without touching walls. | Visual-spatial orientation  Ability for mental rotation |

**Supplementary table 1: Description of exergames played on the Dividat Senso**

|  |  |  |
| --- | --- | --- |
| **Flexi**  **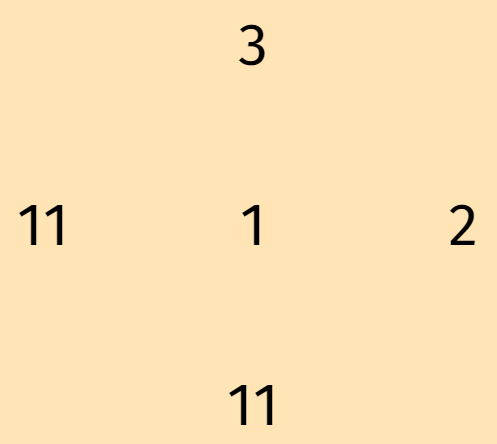**  **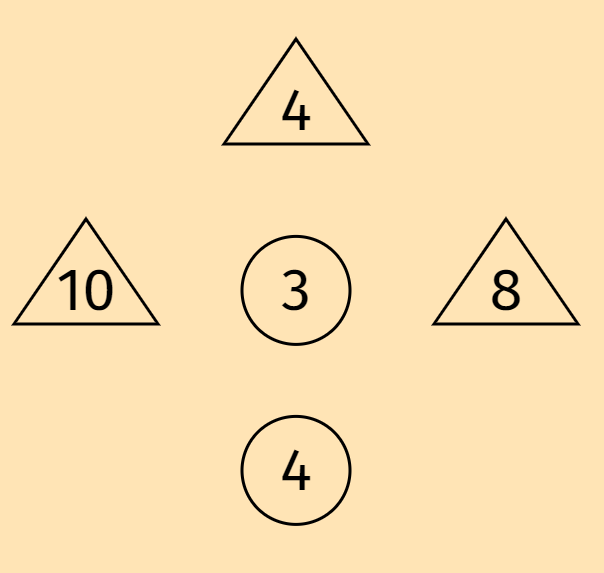** | a) A number is displayed in the center of the screen surrounded by 4 different numbers. The participant has to step in the direction of the next higher number (here: 2).  b) Part b is more challenging: The participant has to step in the direction of the next higher number enclosed by a different shape than in the center. (here: 4 at the top) | Cognitive flexibility |
| **Ski**  **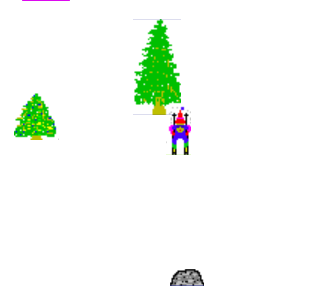** | A little guy skiing down the slopes is displayed on the screen. By weight shifting, the participant can steer to the right or left. The task is to avoid colliding with trees or stones. | Balance control  Weight shifting |
| **Scooper**  **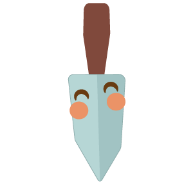** | A carrot appears on the screen. The participant has to place the shovel on top of the carrot to harvest it. The participant can move the shovel by walking to a different spot on the Senso plate. | Precise step execution  Action planning |
| **Simon**  **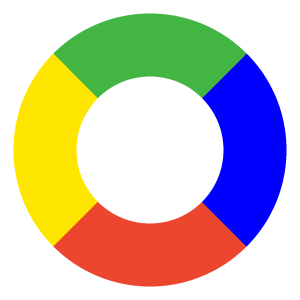** | In the memorization phase, a sequence of acoustic signals is played. Each signal is combined with a colour on the screen. In the subsequent recall phase, the participant has to reproduce the sequence by stepping in the respective direction in the right order. | Short-term memory  Memory span |
| **Snake**  **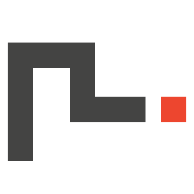** | A white snake moves on the screen. The participant has to steer the snake by stepping in the right direction. The goal is to eat the red apple. | Action planning  Visual-spatial orientation |
| **Tetris**  **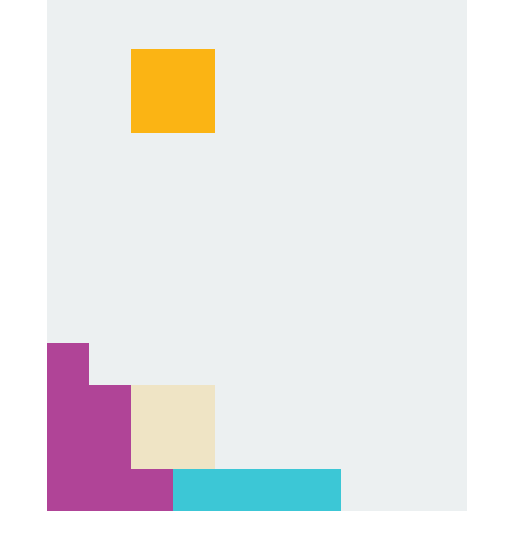** | Different shaped items appear on the top of the screen. The participant has to shift (step right or left) and rotate the items by 90° (forward step). The goal is to place the items as close together as possible. As soon as a row is complete, it disappears. The game is over if a row touches the top of the screen. | Foresighted thinking  Visual-spatial orientation  Ability for mental rotation |
